# Fertilizer placement modulates the effects of soil metabolite formulations on nutrient status, phenylpropanoid–flavonoid metabolism, and sensory quality in flue-cured tobacco

**DOI:** 10.3389/fpls.2026.1949823

**Published:** 2026-09-21

**Authors:** Xu Wei, Hongxun Li, Longchang Wang, Li Zhang, Yechun Lin, Kai Cai, Shengqiang Peng, Jiawen Yuan, Lili Chen, Yihe Sun

**Affiliations:** 1 College of Agronomy and Biotechnology, Southwest University/Engineering Research Center of South Upland Agriculture, Ministry of Education, Chongqing, China; 2 Guizhou Academy of Tobacco Science, Guiyang, China; 3 Anshun Municipal Company of Guizhou Provincial Tobacco Company, Anshun, China; 4 China Tobacco Hebei Industrial Co., Ltd., Shijiazhuang, China

**Keywords:** fertilizer placement, flue-cured tobacco, metabolomics, sensory quality, soil metabolite formulation, transcriptomics, well-cellar application, WGCNA

## Abstract

**Background:**

The quality of flue-cured tobacco depends on nutrient acquisition, carbon–nitrogen allocation, and quality-related secondary metabolism, particularly phenylpropanoid and flavonoid pathways. However, whether fertilizer placement and exogenous soil metabolite formulations can jointly improve leaf nutritional physiology and cured-leaf quality remains unclear.

**Methods:**

A field experiment was conducted with the flue-cured tobacco cultivar Yunyan 87 (*Nicotiana tabacum* L.). Six treatments combined two fertilizer placement methods, strip application and well-cellar application, with two soil metabolite formulations at the same total fertilizer application rate. Nitrogen, phosphorus, and potassium contents and the activities of polyphenol oxidase (PPO), phenylalanine ammonia-lyase (PAL), and sucrose-phosphate synthase (SPS) were determined in mature middle leaves. Sensory evaluation of cured leaves was integrated with transcriptomic, metabolomic, multi-omics, and weighted gene co-expression network analyses.

**Results:**

Compared with conventional strip application (CK1), well-cellar application (CK2) increased leaf nitrogen, phosphorus, and potassium contents by 4.45%, 7.57%, and 6.89%, respectively, with significant increases in nitrogen and potassium. Relative to CK2, A3 (well-cellar application with soil metabolite formulation 1) and A4 (well-cellar application with soil metabolite formulation 2) increased PPO activity by 29.49% and 28.16%, respectively, and PAL activity by 81.08% and 73.91%, respectively, whereas SPS activity showed only a non-significant upward trend. A3, which combined well-cellar application with soil metabolite formulation 1, showed the best overall sensory profile, with improved aroma quality, reduced irritancy and off-odors, and greater smoke softness. Transcriptomic and metabolomic analyses showed that treatment-responsive genes and metabolites were mainly associated with plant hormone signaling, photosynthesis, sugar and amino acid metabolism, and phenylpropanoid and flavonoid biosynthesis. Integrated omics analysis revealed treatment-dependent changes in phenylpropanoid–flavonoid metabolism. Weighted gene co-expression network analysis (WGCNA) identified a darkgreen module containing 4,363 genes that was significantly correlated with several flavonoid-related metabolites, including quercetin, kaempferol, myricetin, and 5,7-dihydroxyflavanone (*r* = 0.56–0.69), and eight candidate hub genes were identified.

**Conclusion:**

Well-cellar application combined with soil metabolite formulation 1 may improve nitrogen and potassium status in mature leaves and coordinate phenylpropanoid–flavonoid metabolism and quality-precursor accumulation, thereby enhancing the overall sensory quality of cured leaves. These findings highlight fertilizer placement as an important determinant of soil metabolite formulation performance and provide a theoretical basis for precision rhizosphere fertilization and the rational use of soil metabolite formulations in flue-cured tobacco production.

## 1 Introduction

The quality of flue-cured tobacco is determined by the coordinated accumulation and transformation of primary and secondary metabolites during plant growth, maturation, and curing. Nitrogen (N) is essential for leaf growth and the synthesis of nitrogen-containing compounds, whereas potassium (K) contributes to osmotic regulation, enzyme activation, assimilate transport, and tobacco quality. However, nutrient availability alone does not fully determine nutrient acquisition or product quality. Fertilizer placement can modify nutrient distribution in the rhizosphere, root–fertilizer contact, and root responses to localized nutrient patches, thereby affecting nutrient uptake and aboveground nutrient accumulation (Nkebiwe et al., 2016). Plants can also adjust root architecture and ion transport in response to spatially heterogeneous nutrient supplies through hormonal regulation (Jia et al., 2022). In flue-cured tobacco, however, the extent to which different fertilizer placement patterns, particularly strip versus well-cellar application, alter leaf metabolism and quality formation remains insufficiently understood.

The rhizosphere provides another potential level of regulation. Low-molecular-weight compounds, including sugars, amino acids, organic acids, alcohols, and phenolic acids, can participate in nutrient mobilization, microbial metabolism, and other rhizosphere processes (Chen et al., 2017; Gomez‐Zepeda et al., 2021; Afridi et al., 2024). These observations provide a rationale for applying exogenous metabolite formulations to modify the rhizosphere environment. However, the effects of such formulations are unlikely to be independent of their spatial context. Exogenous compounds are not equivalent to natural root exudates, and their effects may depend on compound composition, application rate, soil conditions, and fertilizer placement. Thus, fertilizer placement could determine the local concentration, persistence, and accessibility of both nutrients and exogenous metabolites, providing a potential basis for an interaction between fertilizer placement and metabolite formulation. Although localized fertilizer management and exogenous rhizosphere-associated compounds have each been investigated, whether their combination produces treatment-dependent effects on nutrient acquisition and tobacco quality has not been systematically addressed. In particular, it remains unclear whether the well-cellar placement pattern can enhance the response to a metabolite formulation compared with conventional strip application.

Tobacco quality is closely associated with the coordinated metabolism of carbohydrates, amino acids, organic acids, polyphenols, and other precursors before and during curing (Banožić et al., 2020; Liu et al., 2022; Geng et al., 2023; Li et al., 2024). Among these pathways, phenylpropanoid metabolism provides an important biochemical link between primary carbon–nitrogen metabolism and quality-related secondary metabolism. Phenylalanine ammonia-lyase (PAL) initiates phenylpropanoid biosynthesis, which subsequently produces hydroxycinnamic acids, lignin-related precursors, and flavonoids through multiple enzymatic steps. Nutrient conditions can alter both the expression of genes involved in these pathways and the accumulation of associated metabolites in tobacco (Jia et al., 2025). However, nutrient measurements and a limited number of enzyme activities cannot sufficiently explain how fertilizer placement and exogenous metabolite formulations jointly influence metabolic regulation and quality formation. Integrated transcriptomic and metabolomic analyses can therefore provide complementary evidence at the gene-expression and metabolite levels, while weighted gene co-expression network analysis (WGCNA) can be used to identify co-expression patterns associated with target metabolites (Langfelder and Horvath, 2008; Tohge et al., 2015; Savoi et al., 2022).

To address these gaps, we conducted a field experiment using flue-cured tobacco cultivar Yunyan 87 under the same total fertilizer-mixture application rate and compared conventional strip application with well-cellar application. Two soil metabolite formulations with distinct chemical compositions were separately combined with each fertilizer placement method. Formulation 1 mainly contained sugars, organic acids, amino acids, and inositol-related compounds, whereas formulation 2 mainly consisted of amines, organic acids, amino acids and their derivatives, and phenolic acids. We first evaluated leaf N, P, and K contents and the activities of PAL, polyphenol oxidase (PPO), and sucrose-phosphate synthase (SPS), together with the sensory quality of cured middle leaves. We then integrated transcriptomic and metabolomic analyses to investigate treatment-associated changes in carbon–nitrogen metabolism and phenylpropanoid–flavonoid biosynthesis, followed by WGCNA to identify gene co-expression patterns associated with representative metabolites. Specifically, we aimed to determine whether the effects of soil metabolite formulations depended on fertilizer placement and formulation composition, and whether an appropriate combination could improve leaf nutritional status while coordinating metabolic processes related to nutrient use and phenylpropanoid–flavonoid metabolism. This approach was intended to clarify the physiological and molecular basis of fertilizer placement–dependent responses to soil metabolite formulations and to provide a basis for more precise fertilizer management in flue-cured tobacco production.

## 2 Materials and methods

### 2.1 Experimental site and treatment design

The field experiment was conducted at the Yejiaping Village experimental site in Shizi Township, Pingba District, Anshun City, Guizhou Province, China (26°47′N, 106°30′E; 1,610 m above sea level). The flue-cured tobacco cultivar Yunyan 87 (*Nicotiana tabacum* L.), which is widely cultivated in Guizhou Province, was used as the experimental material. The experiment was arranged in a randomized complete block design with six treatments and three replicates per treatment. Each plot covered an area of 60.5 m² and contained 100 tobacco plants, with a row spacing of 1.10 m and a plant spacing of 0.55 m. Two fertilizer placement methods, strip application and well-cellar application, were evaluated in combination with two soil metabolite formulations.

The total application rate of the fertilizer mixture was maintained at 1200 kg ha^-^¹ for all treatments, although the proportions of conventional organic fertilizer and soil metabolite formulation differed among treatments. According to the product specification, the conventional organic fertilizer contained ≥30% organic matter and a guaranteed total nutrient content of N + P_2_O_5_ + K_2_O ≥4.0%. Separate guaranteed contents of N, P_2_O_5_, and K_2_O were not provided by the manufacturer. Therefore, treatment-specific inputs of the individual nutrients could not be reliably calculated from the available product information. Detailed product specifications and the corresponding minimum guaranteed total nutrient inputs are provided in **Supplementary Note S1**. The six treatments were as follows:

CK1: conventional organic fertilizer applied at 1200 kg·ha^-^¹ by strip application;
CK2: conventional organic fertilizer applied at 1200 kg·ha^-^¹ by well-cellar application;
A1: conventional organic fertilizer applied at 1080 kg·ha^-^¹ combined with soil metabolite formulations 1 at 120 kg·ha^-^¹ by strip application;
A2: conventional organic fertilizer applied at 1080 kg·ha^-^¹ combined with soil metabolite formulations 2 at 120 kg·ha^-^¹ by strip application;
A3: conventional organic fertilizer applied at 1080 kg·ha^-^¹ combined with soil metabolite formulations 1 at 120 kg·ha^-^¹ by well-cellar application;
A4: conventional organic fertilizer applied at 1080 kg·ha^-^¹ combined with soil metabolite formulations 2 at 120 kg·ha^-^¹ by well-cellar application.

In treatments A1–A4, the soil metabolite formulations accounted for 10% of the total fertilizer mixture on a weight basis (w/w). Before application, each formulation was thoroughly mixed with the conventional organic fertilizer and applied prior to tobacco transplanting using the designated placement method. Formulation 1 mainly consisted of sugars, amino acids, organic acids, alcohols, and inositol-related compounds, whereas formulation 2 mainly contained amines, organic acids, amino acids and their derivatives, and phenolic acids. The detailed composition and proportions of the two formulations are provided in **Supplementary Table S1**. Except for fertilizer composition and placement method, all treatments received the same field management practices, including transplanting, irrigation, earthing up, pest and disease control, topping and sucker removal, harvesting, and flue-curing, in accordance with the local technical guidelines for flue-cured tobacco production.

### 2.2 Sample collection

When the middle leaves reached natural maturity, three biological replicates were established for each treatment. For each replicate, three plants with uniform growth and no visible symptoms of pests or diseases were randomly selected. Middle leaves at the same stalk position were collected and pooled to form one composite sample. The freshly collected leaves were divided into three portions. The first portion was dried and ground for the determination of nitrogen, phosphorus, and potassium contents. The second portion was immediately frozen in liquid nitrogen and stored at −80 °C for the measurement of polyphenol oxidase (PPO), phenylalanine ammonia-lyase (PAL), and sucrose-phosphate synthase (SPS) activities. The third portion was also immediately frozen in liquid nitrogen and stored at −80 °C for transcriptomic and metabolomic analyses.

### 2.3 Determination of leaf nutrient contents and enzyme activities

Fresh leaf samples used for nutrient and enzyme activity measurements were divided into two portions. One portion was heated at 105 °C for 1 h to terminate metabolic activity and then dried at 80 °C to a constant weight. The dried samples were ground and passed through a 60-mesh sieve. The other portion was used for the determination of enzyme activities. The dried samples were digested with H_2_SO_4_–H_2_O_2_, after which total nitrogen (TN), total phosphorus (TP), and total potassium (TK) contents were determined (Tang et al., 2020). TN was measured using the Kjeldahl method with a KJELTEC™ 8400 analyzer. TP was determined spectrophotometrically at 700 nm according to the Chinese agricultural standard NY/T 85–1988. TK was measured by flame photometry at 766.5 nm according to NY/T 87–1988 (Cui et al., 2022). The activities of PPO, PAL, and SPS in fresh leaves were measured using commercial assay kits supplied by Beijing Solarbio Science & Technology Co., Ltd. (Beijing, China), following the manufacturer’s instructions. Enzyme activities were expressed on a fresh-weight basis as U·g^-^¹ FW.

### 2.4 Sensory evaluation

After flue-curing and grading, C3F-grade tobacco leaves from each treatment were selected for sensory evaluation. Before evaluation, the samples were conditioned under standardized temperature and relative humidity conditions. Sensory evaluation was conducted by five trained panelists using blind-coded samples. Nine sensory attributes were assessed: aroma quality, amount of aroma, concentration, irritancy, offensive odor, physiological strength, aftertaste, softness and fineness, and sweetness. The score for each attribute was calculated as the mean of the scores assigned by the five panelists. Detailed scoring criteria are provided in **Supplementary Table S2**.

### 2.5 Metabolomic analysis

Approximately 100 mg of frozen tobacco leaf tissue was ground with a 6-mm bead and extracted with 800 μL of methanol/water solution (4:1, v/v) containing internal standards. Samples were homogenized at −10 °C and 50 Hz for 6 min, ultrasonicated at 5 °C for 30 min, incubated at −20 °C for 30 min, and centrifuged at 13,000 × *g* and 4 °C for 15 min. The resulting supernatants were collected for LC–MS/MS analysis. A pooled quality-control sample was prepared by mixing equal aliquots of all samples and was injected periodically to monitor analytical stability.

Metabolite profiling was performed using a UHPLC–Orbitrap Exploris 240 system equipped with an ACQUITY BEH C18 column (100 mm × 2.1 mm, 1.7 μm). The mobile phases consisted of 2% acetonitrile in water containing 0.1% formic acid (solvent A) and acetonitrile containing 0.1% formic acid (solvent B). The flow rate was 0.40 mL min^-^¹, the column temperature was 40 °C, and the injection volume was 3 μL. Mass spectra were acquired in both positive- and negative-ion electrospray ionization modes over an *m/z* range of 70–1,050 using data-dependent acquisition.

Raw data were processed using Progenesis QI for peak detection, alignment, and normalization, and metabolites were annotated against the Majorbio plant-specific metabolite database. Features with a relative standard deviation greater than 30% in quality-control samples were excluded. Principal component analysis and orthogonal partial least-squares discriminant analysis were performed using the ropls package in R. Differentially accumulated metabolites were identified using VIP > 1, *P* < 0.05, and |log_2_FC|>1 Kyoto Encyclopedia of Genes and Genomes pathway annotation and enrichment analyses were performed using the scipy.stats package in Python.

### 2.6 Transcriptomic analysis

Total RNA was extracted from fresh tobacco leaves using TRIzol^®^ Reagent according to the manufacturer’s instructions. RNA concentration and purity were determined using a NanoDrop ND-2000 spectrophotometer, and RNA integrity was evaluated using an Agilent 5300 Bioanalyzer. High-quality RNA was used to construct strand-specific mRNA libraries. Poly(A)+ mRNA was enriched using oligo(dT)-conjugated magnetic beads and fragmented, followed by first-strand cDNA synthesis using random hexamer primers and second-strand synthesis with dUTP incorporation. The resulting cDNA was subjected to end repair, 3′ adenylation, adapter ligation, fragment selection, and PCR amplification. Qualified libraries were sequenced on an Illumina NovaSeq X Plus platform using a paired-end 150-bp strategy.

Raw reads were processed using fastp to remove adapter-contaminated and low-quality reads. Clean reads were aligned to the *Nicotiana tabacum* cv. TN90 reference genome (GCF_000715135.1, Ntab-TN90) using HISAT2, and reference-guided transcript assembly was performed using StringTie. Gene expression abundance was estimated using RSEM. Raw gene-level counts were used for differential expression analysis, whereas the FPKM expression matrix was used for visualization and WGCNA.

### 2.7 qRT-PCR Validation

To verify the expression patterns of candidate hub genes identified by RNA-seq, WGCNA, and MCC analyses, eight candidate genes were selected for real-time quantitative reverse transcription PCR (qRT-PCR) analysis. The Actin gene was used as the internal reference. Gene-specific primers were designed based on the corresponding transcript sequences, and their specificity was evaluated using NCBI Primer-BLAST. The primer sequences are listed in **Supplementary Table S4**. qRT-PCR was performed using 2× SYBR^®^ Green MIX (Aidlab, Cat. No. PC3302) on a qTOWER2.2 real-time PCR system (Analytik Jena, Germany). The amplification program consisted of an initial denaturation at 95 °C for 3 min, followed by 40 cycles of denaturation at 95 °C for 10 s and annealing and extension at 60 °C for 30 s. Relative gene expression levels were calculated using the 2^−ΔΔ^*^Ct^* method.

### 2.8 Data analysis

#### 2.8.1 Conventional statistical analysis

Physiological and biochemical data were initially organized using Microsoft Excel 2016 and statistically analyzed using SPSS version 26.0. Data are presented as the mean ± standard deviation (SD) of three biological replicates. Differences among treatments were evaluated using one-way analysis of variance (ANOVA), followed by Duncan’s multiple range test. Differences were considered statistically significant at *P* < 0.05. Different lowercase letters in the figures indicate significant differences among treatments. Physiological and biochemical data were mainly visualized using the ggplot2 package in R, while selected figures were generated using Origin 2026. Sensory evaluation results are presented as the mean scores of individual sensory attributes and were used for descriptive comparisons of sensory quality among treatments.

#### 2.8.2 Metabolomic and transcriptomic data analysis

Principal component analysis (PCA) was performed to evaluate the overall metabolic and transcriptomic profiles of samples from different treatments, and the first two principal components (PC1 and PC2) were used for two-dimensional visualization. Differentially accumulated metabolites (DAMs) were identified using the following criteria: variable importance in projection (VIP) > 1, *P* < 0.05, and |log_2_FC|>1. Differentially expressed genes (DEGs) were identified based on a fold change (FC) > 2 or < 0.5 and *P* < 0.05. Volcano plots were generated using log_2_*FC* as the x-axis and -log10(P-value) as the y-axis, with significantly upregulated, significantly downregulated, and non-significantly changed metabolites or genes indicated by different colors. DAMs and DEGs were separately subjected to Kyoto Encyclopedia of Genes and Genomes (KEGG) pathway annotation and enrichment analysis and were subsequently jointly mapped to the phenylpropanoid and flavonoid biosynthesis pathways to compare treatment-dependent changes in the expression of related genes and the accumulation of associated metabolites. Omics data analyses and visualization were mainly performed in R, and volcano plots were generated using the ggplot2 package. In addition, Pearson correlation analysis was performed between the expression levels of phenylpropanoid–flavonoid pathway-related genes and the abundances of representative metabolites across biological samples to evaluate gene–metabolite associations. The resulting correlation coefficients were visualized as a heatmap (**Supplementary Figure S6**).

Weighted gene co-expression network analysis (WGCNA) was conducted in R using the WGCNA package based on the gene expression matrix expressed as fragments per kilobase of transcript per million mapped reads (FPKM). Genes with low expression levels were removed before network construction. Co-expression modules were identified using the blockwiseModules function with a soft-thresholding power of 15, a minimum module size of 30 genes, and a signed topological overlap matrix (TOM). Modules with similar eigengene profiles were subsequently merged using a module-merging threshold (mergeCutHeight) of 0.25. For each module, the module eigengene (ME), defined as the first principal component of the expression profiles of genes within that module, was calculated. Pearson correlation analysis was then used to assess the relationships between module eigengenes and metabolite traits. The resulting gene co-expression networks were visualized using Cytoscape version 3.8.2 to facilitate interpretation of module–trait relationships.

## 3 Results

### 3.1 Effects of fertilization treatments on nutrient contents and enzyme activities in mature middle leaves

As shown in **Figures 1a–c**, the effects of fertilization treatments on nutrient accumulation in mature middle leaves varied among nutrient elements. Nitrogen (N) and potassium (K) contents showed relatively pronounced responses, whereas phosphorus (P) content differed only numerically among treatments. Compared with conventional strip application (CK1), well-cellar application (CK2) increased leaf N, P, and K contents by 4.45%, 7.57%, and 6.89%, respectively, indicating an overall increase in nutrient accumulation. Under strip application, the addition of soil metabolite formulations produced relatively limited increases in leaf nutrient contents. The N content of A2 was significantly higher than that of CK1, whereas the K contents of A1 and A2 were numerically higher than that of CK1 but remained lower than those of A3 and A4. By contrast, the addition of soil metabolite formulations under well-cellar application had a more pronounced effect on leaf N and K accumulation. The N contents of A3 and A4 were significantly higher than those of CK1, CK2, A1, and A2, with the highest value observed in A3. The K content of A3 was significantly higher than those of CK1, CK2, A1, and A2, whereas no significant difference was detected between A3 and A4. Although P contents were numerically higher in A3 and A4 than in the other treatments, the differences were not significant. Overall, well-cellar application combined with soil metabolite formulations was more favorable for N and K accumulation in mature middle leaves, with A3 showing the best overall performance.

**Figure 1:**
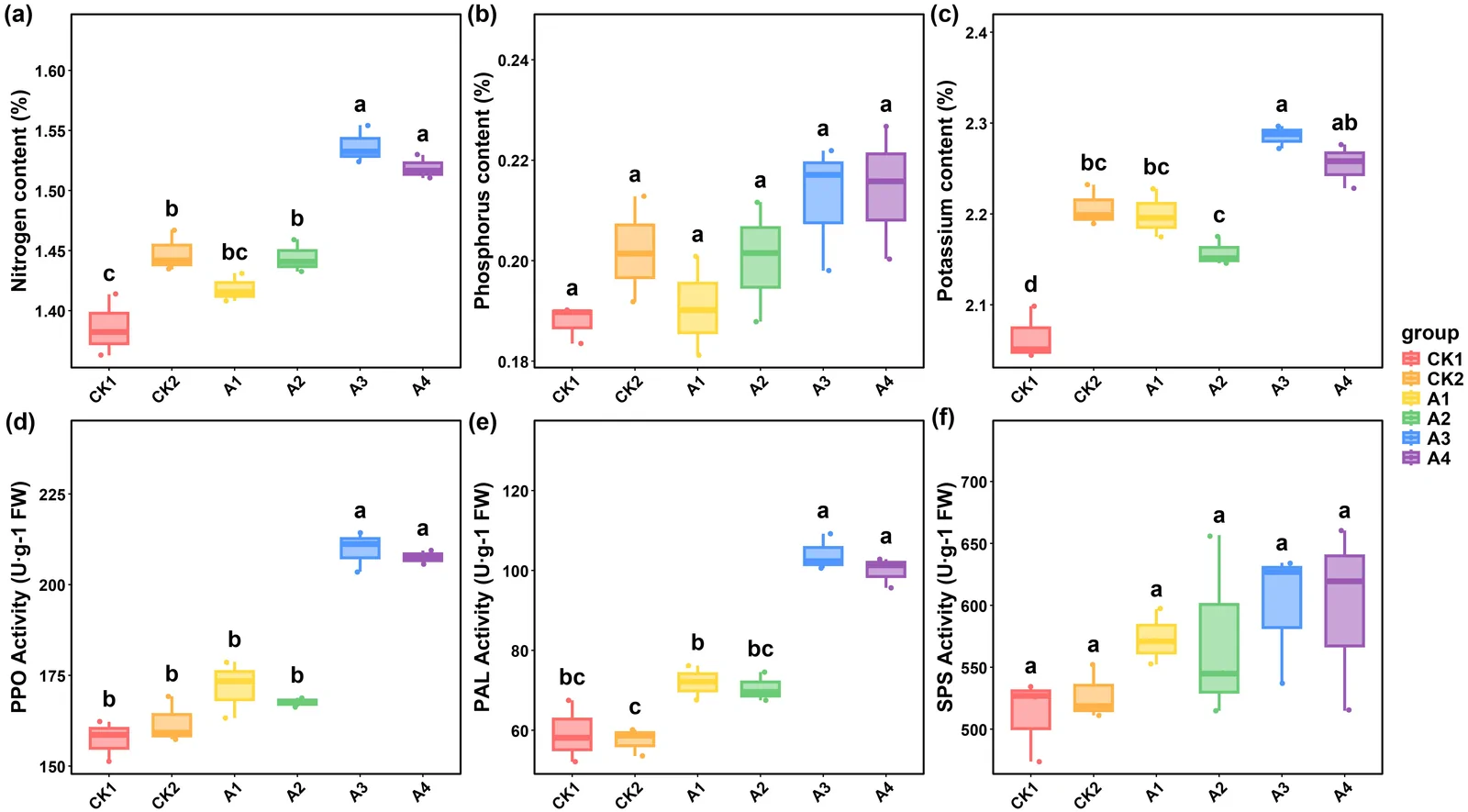
Effects of fertilization method and soil metabolite application on nutrient accumulation and enzyme activities in tobacco middle leaves at maturity. **(a)** Nitrogen content; **(b)** phosphorus content; **(c)** potassium content; **(d)** polyphenol oxidase (PPO) activity; **(e)** phenylalanine ammonia-lyase (PAL) activity; and **(f)** sucrose phosphate synthase (SPS) activity.

As shown in **Figures 1d–f**, the activities of polyphenol oxidase (PPO), phenylalanine ammonia-lyase (PAL), and sucrose-phosphate synthase (SPS) responded differently to the fertilization treatments. Compared with CK1, CK2 slightly increased PPO and SPS activities but slightly decreased PAL activity. Under strip application, PPO activities in A1 and A2 did not differ significantly from those in CK1 and CK2. PAL activity showed an increasing trend, and the value in A1 was significantly higher than that in CK2. Under well-cellar application, the addition of soil metabolite formulations significantly increased PPO and PAL activities. Relative to CK2, PPO activity increased by 29.49% and 28.16% in A3 and A4, respectively, while PAL activity increased by 81.08% and 73.91%, respectively. Both activities were significantly higher in A3 and A4 than in the other treatments. SPS activity was also relatively high in A3 and A4, increasing by 13.68% and 13.45%, respectively, compared with CK2; however, no significant differences were detected among treatments.

### 3.2 Effects of fertilization treatments on the sensory quality of cured middle leaves

As shown in **Figure 2**, the sensory quality scores of cured middle leaves varied among fertilization treatments. Compared with conventional strip application (CK1), well-cellar application (CK2) mainly increased the scores for aroma quality, concentration, offensive odor, and physiological strength. The aroma quality and offensive odor scores increased by 1.67%, whereas the concentration and physiological strength scores increased by 3.45%. The scores for amount of aroma, irritancy, aftertaste, softness and fineness, and sweetness remained largely unchanged. Under strip application, the addition of soil metabolite formulations 1 improved the overall sensory performance of A1 relative to CK1 and CK2. Compared with CK1, A1 increased the scores for aroma quality, amount of aroma, concentration, irritancy, offensive odor, physiological strength, aftertaste, softness and fineness, and sweetness by 3.33%, 1.67%, 5.17%, 1.64%, 3.33%, 3.45%, 3.33%, 1.64%, and 1.67%, respectively. By contrast, the sensory scores of A2 were generally similar to those of CK1 and showed no clear improvement.

**Figure 2:**
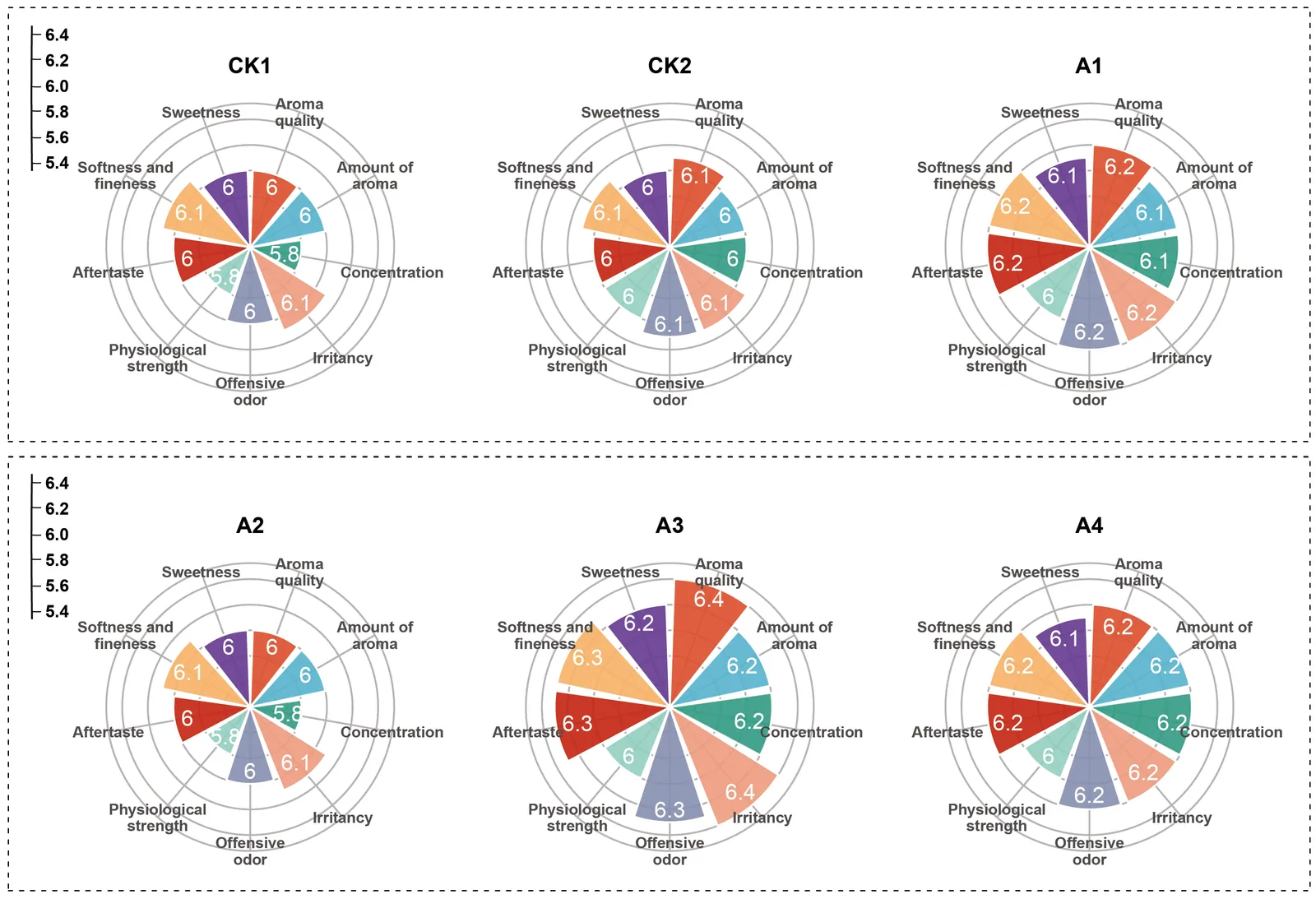
Sensory quality profiles of cured middle tobacco leaves under different fertilization treatments.

Under well-cellar application, the addition of soil metabolite formulations further improved the sensory quality of cured leaves. A3 exhibited the most favorable overall sensory profile based on the mean scores, with scores of 6.4, 6.2, 6.2, 6.4, 6.3, 6.0, 6.3, 6.3, and 6.2 for aroma quality, amount of aroma, concentration, irritancy, offensive odor, physiological strength, aftertaste, softness and fineness, and sweetness, respectively. These values were 6.67%, 3.33%, 6.90%, 4.92%, 5.00%, 3.45%, 5.00%, 3.28%, and 3.33% higher than those of CK1, respectively. Although the overall scores of A4 were slightly lower than those of A3, they remained higher than those of CK1 and CK2, particularly for aroma quality, amount of aroma, concentration, irritancy, offensive odor, aftertaste, and softness and fineness.

### 3.3 Effects of fertilization treatments on the metabolic profiles of fresh middle leaves

As shown in **Figures 3a, b**, the fertilization treatments markedly altered the overall metabolic profiles of fresh middle leaves. Principal component analysis (PCA) showed that PC1 and PC2 explained 30.00% and 15.40% of the total metabolic variation, respectively, accounting for 45.40% in total. Analysis of similarities further confirmed significant differences in metabolite composition among treatments (*R* = 0.5135, *P* = 0.001). CK1 and CK2 were partially separated in the PCA score plot, indicating that fertilizer placement altered the leaf metabolic profile. The addition of soil metabolite formulations resulted in further separation from the corresponding controls. In particular, A3 and A4 showed clear metabolic shifts relative to CK2, whereas A1 and A2 were separated from CK1 to different extents. The sample correlation analysis further showed relatively high correlations among biological replicates within the same treatment and lower correlations among different treatments, indicating that fertilizer placement and soil metabolite formulations jointly influenced the metabolic composition of fresh tobacco leaves.

**Figure 3:**
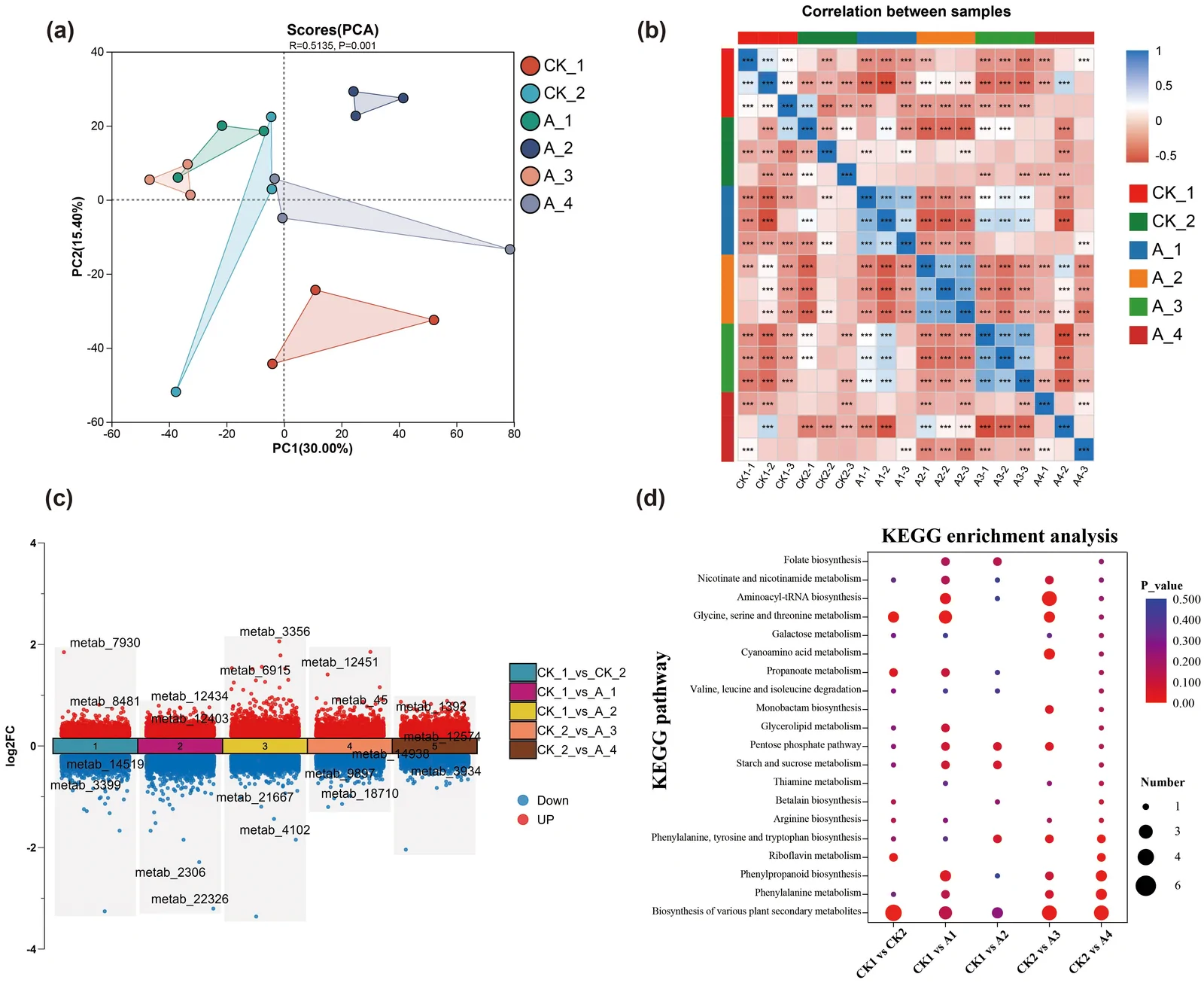
Metabolomic profiling of fresh middle tobacco leaves under different fertilization treatments. **(a)** Principal component analysis (PCA) score plot of metabolomic profiles; **(b)** Pearson correlation heatmap among samples; **(c)** volcano-like plot showing the distribution of up-regulated and down-regulated DAMs across pairwise comparisons; **(d)** KEGG pathway enrichment analysis of DAMs.

Using the thresholds of *P* < 0.05, VIP > 1.0, and |log_2_FC|>1, different numbers of differentially accumulated metabolites (DAMs) were identified among the treatment comparisons (**Figure 3c**). Compared with CK1, CK2 contained 102 up-accumulated and 161 down-accumulated metabolites, indicating that fertilizer placement alone altered metabolite accumulation in tobacco leaves. Under strip application, the addition of soil metabolite formulations resulted in a larger number of DAMs. The CK1 vs. A1 comparison identified 127 up-accumulated and 496 down-accumulated metabolites, whereas the CK1 vs. A2 comparison identified 100 up-accumulated and 416 down-accumulated metabolites. Thus, under strip application, soil metabolite supplementation predominantly reduced the accumulation of a large number of metabolites. Under well-cellar application, the CK2 vs. A3 and CK2 vs. A4 comparisons identified 89 and 50 up-accumulated metabolites and 189 and 160 down-accumulated metabolites, respectively.

KEGG enrichment analysis showed that the DAMs were mainly enriched in the biosynthesis of various plant secondary metabolites, phenylpropanoid biosynthesis, phenylalanine metabolism, starch and sucrose metabolism, the pentose phosphate pathway, glycine, serine and threonine metabolism, and aminoacyl-tRNA biosynthesis (**Figure 3d**). Compared with the corresponding control treatments, the DAMs detected after soil metabolite supplementation, particularly in A3 and A4, were more frequently associated with secondary metabolism, amino acid metabolism, and carbohydrate metabolism.

### 3.4 Effects of fertilization treatments on the transcriptomic profiles of fresh middle leaves

As shown in **Figure 4a**, the transcriptomic profiles of fresh middle leaves differed among fertilization treatments. Principal component analysis (PCA) showed that PC1 and PC2 explained 29.96% and 11.32% of the total variation, respectively, accounting for 41.28% in total. Samples from different treatments were partially separated in the principal component space. CK1 and CK2 were mainly distributed in different regions, indicating that fertilizer placement altered the transcriptomic profile of fresh tobacco leaves. Following the addition of soil metabolite formulations, the treatment groups showed further transcriptional changes relative to their corresponding controls. A1 and A2 were clearly separated from CK1, whereas A3 and A4 also differed from CK2 to varying degrees. Biological replicates from most treatments tended to cluster together, although individual replicates of A1 and CK2 showed relatively high dispersion.

**Figure 4:**
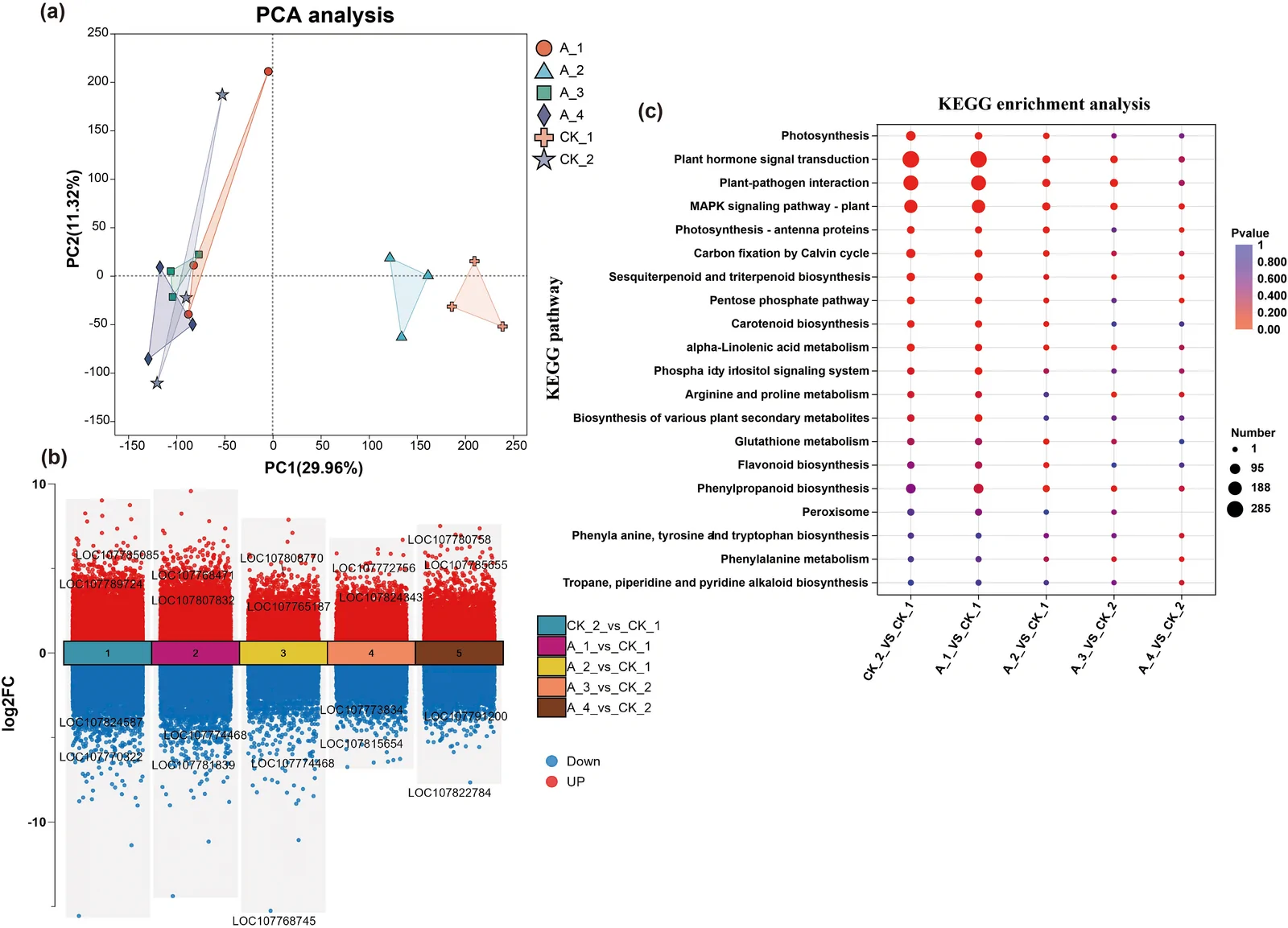
Transcriptomic profiling of fresh middle tobacco leaves under different fertilization treatments. **(a)** Principal component analysis (PCA) score plot of transcriptomic profiles; **(b)** Volcano plot showing the distribution of up-regulated (red) and down-regulated (blue) differentially expressed genes (DEGs) across pairwise comparisons; **(c)** KEGG pathway enrichment analysis of DEGs.

Using the thresholds of FC > 2.0 or FC < 0.5 and *P* < 0.05, different numbers of differentially expressed genes (DEGs) were identified among the treatment comparisons (**Figure 4b**). Compared with CK1, CK2 contained 8,892 DEGs, including 3,734 upregulated and 5,158 downregulated genes, indicating that fertilizer placement alone caused extensive changes in gene expression in fresh middle leaves. Under strip application, A1 induced a strong transcriptional response relative to CK1, with 8,101 DEGs, including 3,617 upregulated and 4,484 downregulated genes. By contrast, A2 induced a weaker transcriptional response, with only 1,407 DEGs, comprising 611 upregulated and 796 downregulated genes. Under well-cellar application, fewer DEGs were identified in A3 and A4 relative to CK2. A3 contained 967 DEGs, including 266 upregulated and 701 downregulated genes, whereas A4 contained 676 DEGs, including 452 upregulated and 224 downregulated genes.

KEGG enrichment analysis showed that the differentially expressed genes (DEGs) were significantly enriched in multiple pathways related to plant growth, development, signaling, primary metabolism, and secondary metabolism, including plant hormone signal transduction, plant–pathogen interaction, the MAPK signaling pathway, photosynthesis, carbon fixation, phenylpropanoid biosynthesis, flavonoid biosynthesis, and the biosynthesis of various plant secondary metabolites (**Figure 4c**). The number of DEGs associated with phenylpropanoid biosynthesis was 77, 78, 27, 15, and 9 across the CK2 vs CK1, A1 vs CK1, A2 vs CK1, A3 vs CK2, and A4 vs CK2 comparisons, respectively. For flavonoid biosynthesis, the corresponding numbers were 32, 32, 11, 1, and 2.

### 3.5 Integrated regulation of phenylpropanoid and flavonoid biosynthesis pathways under different fertilization treatments

Integrated transcriptomic and metabolomic analyses were conducted to further examine the effects of different fertilization treatments on key secondary metabolic pathways in fresh middle tobacco leaves. As shown in **Figure 5**, differentially expressed genes and differentially accumulated metabolites were mainly distributed across nodes related to phenylpropanoid and flavonoid biosynthesis, indicating that these pathways were important components of the response to fertilizer placement and soil metabolite supplementation.

**Figure 5:**
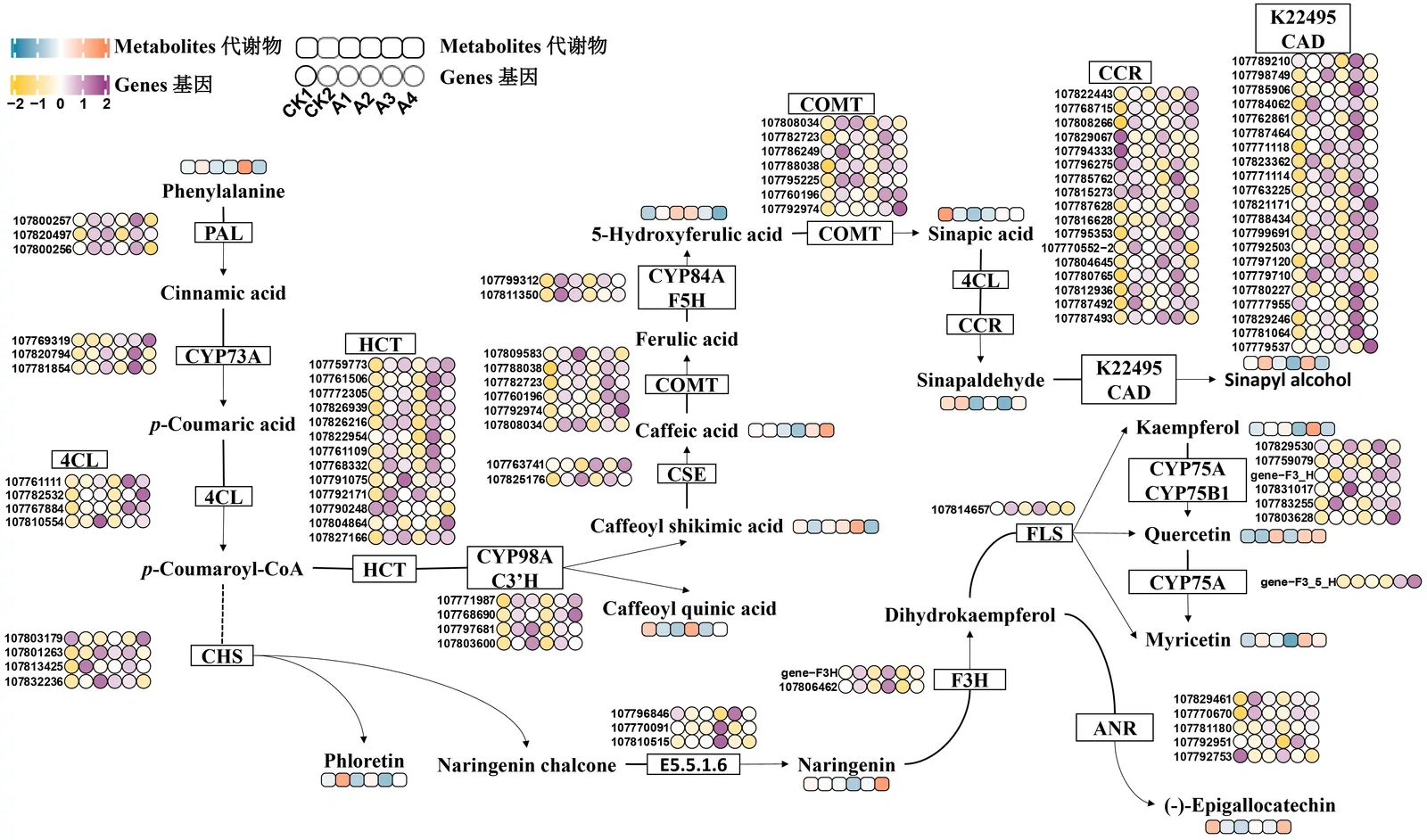
Integrated transcriptomic and metabolomic analysis of phenylpropanoid and flavonoid biosynthesis pathways in fresh middle tobacco leaves under different fertilization treatments. The pathway map shows changes in representative metabolites and genes involved in phenylpropanoid biosynthesis and flavonoid biosynthesis. Squares indicate metabolites, and circles indicate genes. The six columns represent CK1, CK2, A1, A2, A3 and A4, respectively. The color scale represents the relative abundance of metabolites or the relative expression level of genes after normalization.

In the phenylpropanoid biosynthesis pathway, phenylalanine was converted to cinnamic acid by PAL, followed by reactions involving CYP73A, 4CL, HCT, CYP98A, COMT, CCR, and CAD, which participate in the formation of *p*-coumaric acid, caffeic acid, ferulic acid, sinapic acid, and lignin monomer-related precursors. The expression patterns of PAL, CYP73A, 4CL, HCT, CYP98A, COMT, CCR, and CAD varied among treatments. Corresponding metabolites, including caffeoylquinic acid, caffeoylshikimic acid, caffeic acid, ferulic acid, 5-hydroxyferulic acid, sinapic acid, sinapaldehyde, and sinapyl alcohol, also showed differential accumulation among treatments. A3 and A4 exhibited concurrent changes in gene expression and metabolite accumulation at multiple nodes of the phenylpropanoid pathway, suggesting that well-cellar application combined with soil metabolite formulations may further affect hydroxycinnamic acid metabolism and lignin precursor-related processes.

In the flavonoid biosynthesis pathway, after *p*-coumaroyl-CoA entered the downstream branches, CHS, E5.5.1.6, F3H, FLS, CYP75A, CYP75B1, and ANR participated in the formation of naringenin, dihydrokaempferol, kaempferol, quercetin, myricetin, and catechin-related compounds. As shown in **Figure 5**, both the expression of flavonoid biosynthesis-related genes and the accumulation of related metabolites varied among fertilization treatments. Metabolites including naringenin, kaempferol, quercetin, myricetin, phloretin, and epigallocatechin showed differential accumulation patterns among treatments. A3 and A4 showed treatment-dependent changes in gene expression and metabolite accumulation at nodes associated with FLS, CYP75A/CYP75B1, and ANR. Pearson correlation analysis further revealed treatment-associated positive and negative associations between pathway-related genes and representative phenylpropanoid–flavonoid metabolites (**Supplementary Figure S6**), providing additional evidence for coordinated covariation between gene expression and metabolite accumulation.

### 3.6 Identification of key gene modules associated with phenylpropanoid and flavonoid metabolism using WGCNA

Weighted gene co-expression network analysis (WGCNA) was performed using the transcriptomic expression data to identify gene modules associated with phenylpropanoid- and flavonoid-related metabolites. As shown in **Figure 6a**, hierarchical clustering combined with dynamic tree cutting initially identified 79 co-expression modules. After merging modules with similar eigengene profiles using a merge threshold of 0.25, 16 co-expression modules were retained. The darkgreen module contained 4,363 genes.

**Figure 6:**
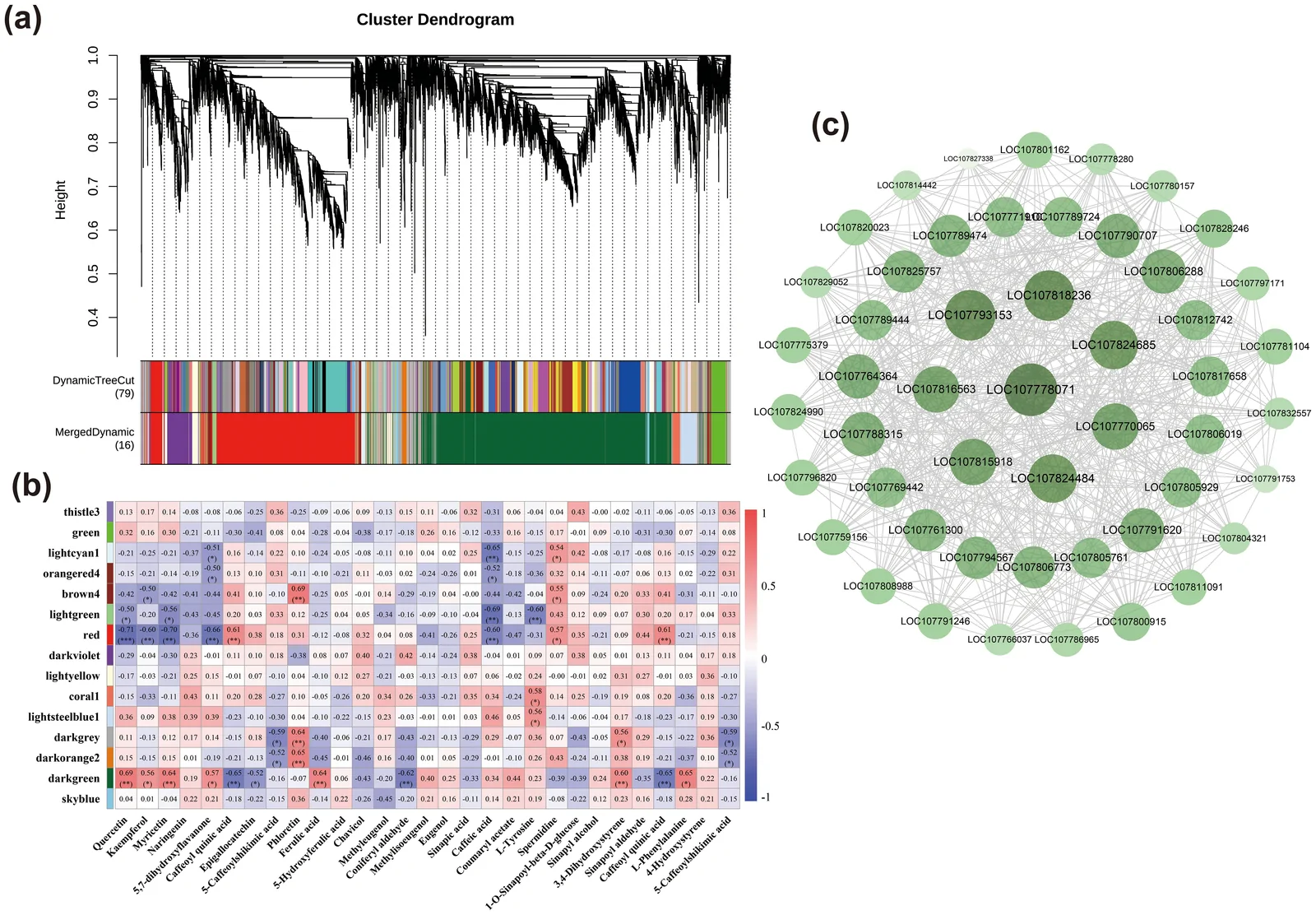
Identification of key gene modules associated with phenylpropanoid and flavonoid metabolism through WGCNA analysis. **(a)** Cluster dendrogram of co-expressed genes and module assignment based on dynamic tree cutting and module merging. **(b)** Heatmap showing correlations between gene modules and representative metabolites involved in phenylpropanoid and flavonoid metabolism. The numbers in each cell indicate Pearson correlation coefficients, and asterisks indicate significance levels. **(c)** Co-expression network of the top 50 hub genes in the darkgreen module identified using the maximal clique centrality (MCC) algorithm in CytoHubba. Node size represents the relative connectivity of each gene within the network.

Module–metabolite correlation analysis showed distinct correlation patterns between the 16 modules and representative phenylpropanoid- and flavonoid-related metabolites (**Figure 6b**). The darkgreen module was positively correlated with quercetin (r = 0.69), kaempferol (r = 0.56), myricetin (r = 0.64), 5,7-dihydroxyflavanone (r = 0.57), and ferulic acid (r = 0.64). Negative correlations were observed between the darkgreen module and caffeoylquinic acid (r = −0.65) and coniferyl aldehyde (r = −0.62). Based on the module–metabolite correlation results, the darkgreen module was selected for subsequent co-expression network analysis.

Genes in the darkgreen module were ranked according to maximal clique centrality (MCC) using the CytoHubba plugin in Cytoscape (**Figure 6c**). Among the highly ranked genes, LOC107776311, LOC107777754, LOC107804321, LOC107812742, LOC107789474, LOC107824990, LOC107814442, and LOC107796072 were located in the core region of the network and were selected as candidate hub genes.

The expression patterns of the eight candidate hub genes were further examined by qRT-PCR and compared with the RNA-seq results (**Supplementary Figure S7**). In the CK2 vs. CK1 and A4 vs. CK2 comparisons, all eight genes showed the same direction of expression change between qRT-PCR and RNA-seq. In the A1 vs. CK1 comparison, seven of the eight genes showed consistent expression directions. In the A2 vs. CK1 and A3 vs. CK2 comparisons, five of the eight genes showed consistent expression directions between the two methods. Differences in fold-change magnitude were also observed for individual genes.

## 4 Discussion

### 4.1 Effects of fertilization treatments on nutrient accumulation and enzyme activities in mature middle leaves

Compared with conventional strip application (CK1), well-cellar application (CK2) increased N, P, and K contents in mature middle leaves, with significant increases observed for N and K. This finding indicates that altering fertilizer placement may improve the uptake and accumulation of major nutrients in tobacco plants. When soil metabolite formulations were combined with well-cellar application, leaf N content increased further in both A3 and A4. In addition, K content was significantly higher in A3 than in CK2, while A4 also showed an increasing trend, indicating that different combinations of fertilizer placement and soil metabolite formulations produced distinct nutrient responses. Tobacco is a typical K-demanding crop. Potassium is involved in photosynthate transport, osmotic regulation, and the activation of multiple enzymes, and is closely associated with leaf combustibility, aroma characteristics, and industrial usability. Insufficient K is considered an important constraint on improving flue-cured tobacco quality in some production regions (Hu et al., 2021). Nitrogen directly affects vegetative growth, leaf development, and carbon–nitrogen balance during maturation. Appropriate N accumulation can help maintain leaf physiological activity, whereas excessive N supply may promote the accumulation of nitrogenous compounds and adversely affect irritancy and overall sensory balance (Ren et al., 2025; Yang et al., 2025). Therefore, the higher N and K contents observed in A3 and A4 may have provided a nutritional basis for maintaining active metabolism in mature leaves. However, whether these changes also promote plant growth and improve the chemical balance of cured leaves requires further validation using plant height, leaf area, dry matter accumulation, yield, and routine chemical composition data. Although P content was numerically higher in A3 and A4, the differences among treatments were not significant, suggesting that P was less responsive than N and K. This weaker response may be related to the soil P supply, the P demand of tobacco plants at maturity, and P redistribution among plant organs.

Rhizosphere nutrient availability is jointly influenced by fertilizer placement, root distribution, soil pH, organic acid release, and microbial activity. Low-molecular-weight organic acids can alter mineral nutrient availability through acidification, ion complexation, and effects on microbial metabolism (Jones, 1998; Rengel and Marschner, 2005; Ma et al., 2022). Trehalose, mannose, inositol, alanine, succinic acid, and lactic acid in formulation 1 may influence rhizosphere carbon availability, nutrient transformation, and plant metabolism. In contrast, *n*-butylamine, pyroglutamic acid, leucine, and syringic acid in formulation 2 may act through processes associated with nitrogen-containing compounds and phenolic acids. Both formulations contained succinic acid, which may partly explain the nutrient responses observed in both A3 and A4. Previous studies have shown that low-molecular-weight soil metabolites, including succinic acid, can affect soil chemical properties, microbial communities, and metabolic profiles, thereby altering the chemical composition of tobacco leaves (Li et al., 2025).

A3 and A4 also significantly increased PAL and PPO activities, suggesting that the application of soil metabolite formulations under well-cellar fertilization may enhance the potential for phenylalanine flux into phenolic secondary metabolism and increase the oxidative transformation of phenolic compounds. PAL is the entry enzyme of phenylpropanoid biosynthesis and contributes to the formation of phenolic acids, flavonoids, and lignin precursors. PPO participates in the oxidation of phenolic compounds and may affect color and flavor development during tobacco maturation and curing, although its effect on quality depends on the balance between substrate availability and the extent of oxidation (Sharma et al., 2019; Zhao et al., 2023). SPS activity in A3 and A4 showed only a non-significant upward trend and should therefore be regarded as supporting, rather than conclusive, evidence of altered sugar metabolism. Taken together, A3 showed favorable performance in N and K accumulation and in PAL and PPO activities, suggesting that soil metabolite formulations 1 combined with well-cellar application may be more effective in maintaining leaf nutritional status and secondary metabolic activity at maturity, thereby providing a physiological basis for the formation of quality-related precursors.

An important consideration is that the treatments were standardized by total fertilizer-mixture mass rather than by strictly equivalent nutrient inputs. According to the product specification, the conventional organic fertilizer used in this study had a guaranteed total nutrient content of N + P_2_O_5_ + K_2_O ≥4.0% and was applied at 1200 kg ha^-^¹ in CK1 and CK2 but at 1080 kg ha^-^¹ in A1–A4. Accordingly, the conventional organic fertilizer supplied minimum guaranteed total nutrient inputs of 48.0 and 43.2 kg ha^-^¹ of N + P_2_O_5_ + K_2_O, respectively. Because the manufacturer did not provide separate guaranteed contents of N, P_2_O_5_, and K_2_O, treatment-specific inputs of the individual nutrients could not be reliably quantified. In addition, the soil metabolite formulations differed in chemical composition and may also have influenced nutrient availability and plant metabolism. Therefore, differences in nutrient supply among treatments cannot be completely excluded as a potential confounding factor when interpreting the observed physiological and metabolic responses. Future experiments should preferably balance individual N, P, and K inputs across treatments to more precisely distinguish the effects of fertilizer placement from those of soil metabolite formulation composition.

### 4.2 Effects of fertilization treatments on the sensory quality of cured middle leaves

The sensory quality of cured tobacco leaves is a complex trait that develops from the nutritional and maturity status established in the field and the coordinated transformation of sugars, amino acids, organic acids, phenolic compounds, and other aroma precursors during curing. Tobacco quality does not improve through the unidirectional increase of a single nutrient or chemical component but depends on the balance among N, K, sugars, alkaloids, and multiple quality-related precursors. Appropriate K supply promotes assimilate transport, leaf maturation, and combustibility; however, improvements in aroma, aftertaste, and smoke comfort can occur only when nutrient uptake is further translated into a balanced composition of quality precursors (Hu et al., 2021; Zhang et al., 2024). Compared with CK1, CK2 mainly increased the scores for aroma quality, concentration, offensive odor, and physiological strength. The concentration score increased from 5.8 to 6.0, indicating a shift from relatively strong smoke concentration toward a more moderate level. The higher offensive odor score indicated fewer off-notes and improved smoke cleanliness and comfort. By contrast, the scores for amount of aroma, irritancy, aftertaste, softness and fineness, and sweetness remained largely unchanged. These results suggest that changing the placement of organic fertilizer alone improved several sensory attributes but did not substantially alter the overall sensory profile. Fertilizer management may also affect aroma-precursor accumulation and the sensory performance of cured leaves by modifying rhizosphere nutrient transformation, microbial metabolism, and plant nutritional status (Yan et al., 2020). Therefore, the sensory improvement observed in CK2 may have resulted mainly from changes in nutrient availability within the root zone, whereas fertilizer placement alone appeared to have a limited effect on the coordinated regulation of multiple aroma precursors and chemical constituents.

The effects of soil metabolite supplementation varied according to both formulation composition and fertilizer placement. Under strip application, the sensory scores of A1 were generally higher than those of CK1, whereas A2 showed a sensory profile similar to that of CK1. This contrast indicates that formulation 1 had some potential to improve cured-leaf quality under strip application, whereas formulation 2 did not produce a comparable response. Under well-cellar application, both A3 and A4 showed higher overall sensory scores than the corresponding CK2 control, with A3 exhibiting the best overall performance. According to the sensory scoring criteria, A3 obtained an aroma quality score of 6.4, corresponding to a “good” rating. Its irritancy score of 6.4 indicated relatively mild irritation, while its offensive odor score of 6.3 reflected cleaner and more comfortable smoke. The softness and fineness score also reached 6.3, corresponding to a “good” rating. The amount of aroma and concentration scores were both 6.2, indicating relatively abundant aroma and a comparatively moderate smoke concentration. A3 was the only treatment with scores ranging from 6.3 to 6.5 for several attributes, including aroma quality, irritancy, offensive odor, aftertaste, and softness and fineness. Its advantage was therefore mainly reflected in improved aroma quality, reduced irritancy and off-notes, and enhanced smoke softness rather than a simple increase in smoke concentration or physiological strength.

Sugars, free amino acids, Amadori compounds, and phenolic substances in tobacco leaves can undergo Maillard reactions, thermal degradation, and condensation during curing and combustion, generating aldehydes, ketones, and furans associated with sweet, roasted, floral, and fruity aroma characteristics. Coordinated changes among multiple precursors generally provide a better explanation for differences in overall sensory quality than changes in any single component (Liu et al., 2022; Li et al., 2024). Trehalose, mannose, alanine, succinic acid, lactic acid, and inositol in formulation 1 may have indirectly altered the pre-curing accumulation pattern of quality precursors by affecting nutrient availability, rhizosphere carbon supply, and plant carbon–nitrogen metabolism. The improved aroma quality, aftertaste, and softness and fineness observed in A3 may therefore be associated with its higher N and K contents, increased PAL and PPO activities, and treatment-related changes in sugar metabolism and phenolic secondary metabolism.

### 4.3 Integrated regulation of the transcriptomic–metabolic network in fresh middle leaves under different fertilization treatments

The contrasting transcriptomic, metabolomic, physiological, and sensory responses among treatments indicate that the biological effectiveness of a fertilization strategy cannot be inferred simply from the magnitude of molecular reprogramming. In the present study, A1 generated 8,101 DEGs, whereas A3 generated only 967 DEGs, despite both treatments receiving soil metabolite formulation 1. This marked difference indicates that fertilizer placement strongly influenced the overall transcriptional response to the same formulation. The broader response in A1 suggests extensive adjustment of gene expression under strip application, whereas the more limited response in A3 indicates a lower overall degree of transcriptional reprogramming under well-cellar application.

Importantly, a smaller transcriptomic response did not correspond to a weaker physiological or quality response. A3 showed more favorable N and K accumulation, higher PAL and PPO activities, distinct metabolic remodeling, and the best overall sensory performance. The apparent discrepancy between molecular response magnitude and treatment performance may reflect the multilayered regulation of plant metabolism, in which transcript abundance represents only one level of control. Metabolite accumulation is additionally influenced by enzyme activity, substrate availability, post-translational regulation, transport, compartmentalization, competing metabolic branches, and degradation (Tohge et al., 2015; Savoi et al., 2022). Therefore, extensive transcriptional changes do not necessarily translate into greater metabolic or physiological benefits, whereas a comparatively smaller transcriptional response can still be accompanied by substantial changes in enzyme activity and metabolite composition.

The comparison between A1 and A3 further illustrates this distinction. Although A1 contained more phenylpropanoid- and flavonoid-related DEGs in absolute terms than A3, these genes represented only a small fraction of the total transcriptional response. For phenylpropanoid biosynthesis, 78 DEGs were identified in A1 compared with 15 in A3, corresponding to approximately 0.96% and 1.55% of the total DEGs, respectively. Thus, the phenylpropanoid-related response was not absent from A3 and represented a comparable or slightly greater fraction of its smaller DEG set. In contrast, flavonoid biosynthesis involved 32 DEGs in A1 and only 1 in A3, indicating that the superior performance of A3 cannot be attributed to stronger transcriptional activation of the flavonoid pathway. Rather, the favorable phenotype of A3 appears to arise from the combined effects of nutritional status, enzyme activity, primary metabolism, secondary metabolism, and metabolite reorganization.

The pathway-level responses provide a mechanistic framework for this integrated effect. Plant hormone signaling and MAPK pathways can connect changes in the nutritional environment with root development, nutrient acquisition, and downstream metabolic regulation. Auxin-related transcriptional regulation has been associated with root growth and K acquisition in tobacco (Jia et al., 2022; Guan et al., 2025), while MAPK cascades can transmit nutritional and environmental signals to networks involved in growth and metabolism (Jagodzik et al., 2018). Photosynthesis and carbon fixation determine the availability of assimilated carbon, whereas starch and sucrose metabolism regulates carbon storage and assimilate distribution (MacNeill et al., 2017; Zheng et al., 2025). The pentose phosphate pathway provides reducing power and carbon skeletons for aromatic amino acid and phenolic compound biosynthesis, thereby linking carbon metabolism with nitrogen assimilation and phenylpropanoid metabolism (Kruger and Von Schaewen, 2003). These processes may have contributed to the favorable metabolic background observed in A3, in which nutrient status and primary metabolism were maintained alongside changes in quality-related secondary metabolism.

Phenylpropanoid metabolism provides an important biochemical connection between these primary processes and quality-related metabolite formation. PAL initiates the conversion of phenylalanine into cinnamic acid, after which CYP73A and 4CL channel intermediates toward hydroxycinnamic acids, lignin-related compounds, and flavonoids. Nutrient availability can affect both the expression of phenylpropanoid-related genes and the accumulation of associated metabolites in tobacco (Jia et al., 2025). In the present study, the increased PAL activity observed in A3 and A4 was accompanied by changes in PAL, CYP73A, 4CL, HCT, CYP98A, and COMT, together with changes in caffeoylquinic acid, caffeoylshikimic acid, caffeic acid, ferulic acid, 5-hydroxyferulic acid, and sinapic acid. PAL is a key entry point into phenylpropanoid metabolism, and its regulation is closely linked to polyphenol accumulation in tobacco (Shi et al., 2022). NtERF4, for example, has been reported to influence chlorogenic acid and flavonoid biosynthesis through PAL-related regulation (He et al., 2024). These observations suggest that the improved physiological status under A3 was accompanied by regulation of phenylpropanoid metabolism at both enzymatic and molecular levels.

The downstream flavonoid branch further contributes to the formation of quality-related metabolites. CHS directs p-coumaroyl-CoA into flavonoid biosynthesis, while F3H, FLS, CYP75A, CYP75B1, and ANR contribute to the formation and modification of different flavonoid subclasses. F3H and FLS participate in flavonoid skeleton modification and compositional diversification (Wang et al., 2019). In the present study, naringenin, kaempferol, quercetin, myricetin, phloretin, and epigallocatechin showed treatment-dependent changes, indicating that fertilization altered the composition of phenylpropanoid- and flavonoid-derived metabolites rather than simply changing total flavonoid abundance. Previous studies have also shown that flavonoid biosynthesis in tobacco is influenced by both nutrient conditions and developmental stage (Jia et al., 2025; Tong et al., 2025). Because phenolic compounds and flavonoids contribute to antioxidant defense and can participate in biochemical transformations during curing and combustion, their altered accumulation may contribute to differences in the quality of cured tobacco leaves (Liu et al., 2022; Li et al., 2024).

Taken together, the results support the interpretation that the favorable performance of A3 was associated with coordinated regulation across multiple biological levels rather than with the magnitude of transcriptomic change alone. Well-cellar application combined with formulation 1 produced a relatively moderate transcriptional response but was accompanied by favorable N and K status, enhanced PAL and PPO activities, metabolic reorganization, and improved sensory quality. This pattern suggests that fertilizer placement may influence how an applied metabolite formulation is translated into downstream physiological and metabolic responses. However, the present study does not establish a direct causal relationship between individual genes or metabolites and specific sensory attributes, because omics analyses were performed on fresh leaves whereas sensory evaluation was conducted after curing. Further studies integrating rhizosphere measurements, volatile aroma profiling, isotope tracing or metabolic flux analysis, and functional validation will be needed to clarify the mechanisms underlying the superior performance of A3.

### 4.4 WGCNA reveals key co-expression modules and candidate hub genes associated with phenylpropanoid and flavonoid metabolism

WGCNA provided a network-level perspective for interpreting the transcriptional responses associated with phenylpropanoid and flavonoid metabolism under different fertilization treatments. Among the 16 co-expression modules obtained after module merging, the darkgreen module contained 4,363 genes and showed relatively strong correlations with several flavonoid-related metabolites, including quercetin, kaempferol, myricetin, and 5,7-dihydroxyflavanone. Quercetin, kaempferol, and myricetin are important flavonol products derived from the flavonoid biosynthetic pathway, and their simultaneous positive correlations with the same module indicate that the overall expression pattern of genes in the darkgreen module was closely associated with variation in multiple flavonoid components. Previous studies have shown that flavonoid accumulation is jointly influenced by structural genes, transcription factors, transport-related genes, and signaling components. WGCNA is therefore useful for identifying groups of co-expressed genes whose collective expression patterns covary with specific metabolite traits (Ye et al., 2021; Xiong et al., 2023; Arshad et al., 2025; Jia et al., 2025).

The correlation pattern of the darkgreen module also reflected the branched nature of phenylpropanoid metabolism. In addition to its positive correlations with flavonoid metabolites, the module was positively correlated with ferulic acid, but negatively correlated with caffeoylquinic acid and coniferyl aldehyde. Phenylpropanoid metabolism produces multiple classes of secondary metabolites, including hydroxycinnamic acids, lignin-related intermediates, and flavonoids, which share common upstream precursors but diverge into different downstream branches. Therefore, the opposite correlations observed between the darkgreen module and different phenylpropanoid-derived metabolites may reflect differences in the relative allocation of metabolic flux among these branches rather than a uniform activation or suppression of the entire pathway. This interpretation is consistent with the integrated transcriptomic and metabolomic analysis, in which treatment-dependent changes were detected at multiple nodes of phenylpropanoid and flavonoid biosynthesis, including genes associated with PAL, 4CL, HCT, CYP98A, FLS, CYP75A/CYP75B1, and ANR, together with corresponding changes in phenolic acids and flavonoid metabolites.

Although the darkgreen module showed clear associations with several target metabolites, its relatively large gene number indicates that it is more appropriate to interpret it as a broad co-expression program associated with phenylpropanoid–flavonoid metabolic variation rather than as a pathway-specific regulatory module. Module eigengene–metabolite correlations reflect coordinated variation at the network level, but they do not establish direct regulatory relationships between individual genes and metabolites. Accordingly, the biological significance of the module should be interpreted together with pathway annotation, gene connectivity, and gene–metabolite relationships.

Within the darkgreen module, LOC107776311, LOC107777754, LOC107804321, LOC107812742, LOC107789474, LOC107824990, LOC107814442, and LOC107796072 were located in the highly connected region of the MCC-ranked network. Their central positions indicate that their expression patterns were closely connected with those of other genes within the module. However, network centrality alone cannot demonstrate a direct role in phenylpropanoid or flavonoid biosynthesis. Because these genes were identified mainly on the basis of co-expression topology and currently lack direct functional and genetic evidence, they should be regarded as candidate hub genes rather than confirmed regulators. Further integration of functional annotation, module membership, gene significance, and gene–metabolite correlations will be necessary to clarify their potential roles in these metabolic processes.

The qRT-PCR analysis provided additional support for the RNA-seq expression patterns of the candidate genes, although the degree of agreement varied among treatment comparisons. All eight candidate genes showed the same direction of expression change between RNA-seq and qRT-PCR in the CK2 vs. CK1 and A4 vs. CK2 comparisons, and seven of the eight genes showed consistent directions in the A1 vs. CK1 comparison. In the A2 vs. CK1 and A3 vs. CK2 comparisons, five of the eight genes showed consistent directions between the two methods, while differences in fold-change magnitude were also observed for several genes. Such discrepancies may be related to methodological differences between sequencing- and PCR-based quantification, biological variation among samples, or relatively small expression changes for individual genes. Therefore, the qRT-PCR results support the overall RNA-seq expression trends but should not be interpreted as complete validation of every candidate gene under all treatment comparisons.

In combination with the physiological and multi-omics results, the WGCNA analysis further indicates that the responses to fertilization treatments involved coordinated changes in nutrient status and phenylpropanoid–flavonoid metabolism. A3 showed favorable responses in N and K accumulation, PAL and PPO activities, phenylpropanoid–flavonoid metabolism, and cured-leaf sensory quality, whereas its number of DEGs was lower than that observed in several other treatment comparisons. This pattern suggests that the favorable performance of A3 may be associated more with coordinated adjustment of nutrient metabolism and quality-related secondary metabolism than with the magnitude of transcriptional disturbance. Further studies integrating functional annotation, gene–metabolite association analysis, and experimental validation through gene overexpression or silencing are required to determine the specific functions of these candidate hub genes in phenylpropanoid and flavonoid metabolism.

## 5 Conclusion

This study demonstrated that fertilizer placement strongly influenced the responses of flue-cured tobacco to soil metabolite formulations at physiological, metabolic, and sensory levels. Compared with conventional strip application, well-cellar application was more favorable for N and K accumulation in mature middle leaves, and the addition of soil metabolite formulations further enhanced several physiological responses. In particular, A3 and A4 significantly increased PAL and PPO activities relative to CK2, while A3 showed the most favorable overall sensory profile after curing, with improvements in aroma quality, irritancy, offensive odor, aftertaste, and softness and fineness. These results indicate that the combination of well-cellar application and soil metabolite formulation 1 was the most favorable treatment tested for coordinating leaf nutritional status, phenolic metabolism, and cured-leaf sensory quality.

Integrated transcriptomic and metabolomic analyses showed that fertilizer placement and soil metabolite supplementation affected multiple pathways related to plant signaling, photosynthesis, carbon metabolism, amino acid metabolism, and secondary metabolism. Phenylpropanoid and flavonoid biosynthesis represented important components of these responses, with treatment-dependent changes detected in both gene expression and metabolite accumulation at multiple pathway nodes involving PAL, CYP73A, 4CL, HCT, CYP98A, COMT, FLS, CYP75A/CYP75B1, and ANR. Notably, A3 produced a relatively limited transcriptomic response compared with several other treatment comparisons, yet still exhibited favorable nutrient accumulation, enzyme activities, metabolic remodeling, and sensory quality. This pattern suggests that treatment performance depended more on the coordinated adjustment of key physiological and metabolic processes than on the overall magnitude of transcriptional reprogramming.

WGCNA further identified a darkgreen co-expression module containing 4,363 genes that was associated with several phenylpropanoid- and flavonoid-related metabolites, including quercetin, kaempferol, myricetin, 5,7-dihydroxyflavanone, ferulic acid, caffeoylquinic acid, and coniferyl aldehyde. Eight genes, LOC107776311, LOC107777754, LOC107804321, LOC107812742, LOC107789474, LOC107824990, LOC107814442, and LOC107796072, were selected as candidate hub genes based on network centrality, and qRT-PCR generally supported their RNA-seq expression trends. These genes provide candidates for further investigation of gene networks associated with phenylpropanoid–flavonoid metabolic variation.

Overall, the results support the use of well-cellar application combined with soil metabolite formulation 1 as a promising fertilization strategy for improving the nutritional status and sensory quality of flue-cured tobacco through coordinated regulation of primary and secondary metabolism. However, the present study was conducted under unequal guaranteed nutrient inputs between the conventional fertilizer controls and formulation treatments, and the omics analyses were performed on fresh leaves whereas sensory evaluation was conducted after curing. Therefore, direct causal links among rhizosphere processes, candidate genes, metabolite changes, and specific sensory attributes remain to be established. Future studies should balance total nutrient inputs among treatments and integrate soil–root measurements, volatile aroma profiling, metabolic flux analysis, and functional validation of candidate hub genes to clarify the mechanisms underlying the favorable performance of A3.

## Data Availability

The original contributions presented in the study are publicly available. The RNA-seq data generated in this study have been deposited in the NCBI Sequence Read Archive (SRA) database under BioProject accession number PRJNA1529886.
