# Supplementary material for: Fertilizer placement modulates the effects of soil metabolite formulations on nutrient status, phenylpropanoid–flavonoid metabolism, and sensory quality in flue-cured tobacco
Source: Front Plant Sci. 2026 Sep 21;17:1949823. doi: 10.3389/fpls.2026.1949823 (PMC13634966; doi:10.3389/fpls.2026.1949823)
Supplement: Supplementary file 1 [file SupplementaryFile1.docx]

**Types of paper:** Original Research

**Fertilizer placement modulates the effects of soil metabolite formulations on nutrient status, phenylpropanoid–flavonoid metabolism, and sensory quality in flue-cured tobacco**

Xu Wei^1^, Hongxun Li^2*^, Longchang Wang^1^, Li Zhang^3^, Kai Cai^2^, Shengqiang Peng^4^, Jiawen Yuan^3^, Lili Chen^2^, Yihe Sun^2^

**Affiliations:**

1 College of Agronomy and Biotechnology, Southwest University / Engineering Research Center of South Upland Agriculture, Ministry of Education, Chongqing, 400715, China

2 Guizhou Academy of Tobacco Science, Guiyang, 550081, China

3 Anshun Municipal Company of Guizhou Provincial Tobacco Company, Anshun, 561000, China

4 China Tobacco Hebei Industrial Co., Ltd., Shijiazhuang, 050051, China

**^⁎^Corresponding authors:**

Emails: hxli2023@163.com (Hongxun Li)

**Supplementary**

**Note S1** Product specification of the charcoal-based organic fertilizer used in the field experiment

**Table S1** Composition and application proportions of the soil metabolite formulations used in this study

**Table S2** Individual indicator scoring scale for sensory quality characteristics of tobacco leaves

**Table S3** Mean sensory scores and standard deviations of cured middle tobacco leaves under different fertilization treatments.

**Table S4** Primers used for qRT-PCR validation

**Fig. S1** Numbers of differentially accumulated metabolites in different fertilization treatment comparisons

**Fig. S2** Venn diagram showing shared and unique differentially accumulated metabolites among different fertilization treatment comparisons

**Fig. S3** Venn diagram showing shared and unique differentially expressed genes among different fertilization treatment comparisons

**Fig. S4** Selection of the soft-thresholding power for WGCNA network construction

**Fig. S5** Heatmap of the gene co-expression network based on the topological overlap matrix

**Fig. S6** Pearson correlation heatmap of phenylpropanoid–flavonoid pathway-related genes and metabolites in fresh middle tobacco leaves under different fertilization treatments.

**Fig. S7** qRT-PCR validation of selected candidate hub genes

**Note S1. Product specification of the organic fertilizer used in the field experiment.**

The conventional organic fertilizer used in this study contained ≥30% organic matter and had a guaranteed total nutrient content of N + P₂O₅ + K₂O ≥4.0%, according to the product specification. The product specification did not provide separate guaranteed contents for N, P₂O₅, and K₂O. Therefore, separate treatment-specific inputs of N, P₂O₅, and K₂O could not be reliably calculated.

At an application rate of 1200 kg ha⁻¹, the conventional organic fertilizer supplied at least 48.0 kg ha⁻¹ of total N + P₂O₅ + K₂O in CK1 and CK2. In A1–A4, the conventional organic fertilizer was applied at 1080 kg ha⁻¹ and supplied at least 43.2 kg ha⁻¹ of total N + P₂O₅ + K₂O, while the remaining 120 kg ha⁻¹ consisted of the corresponding soil metabolite formulation.

**Table S1** Composition and application proportions of the soil metabolite formulations used in this study

| Formulation | Component | Mass fraction of total mixture applied (%) | Actual application rate (kg·ha⁻¹) |
| --- | --- | --- | --- |
| Formulation 1 | Trehalose | 3.0 | 36.0 |
|  | L-Alanine | 0.5 | 6.0 |
|  | Succinic acid | 2.0 | 24.0 |
|  | L-Lactic acid | 1.0 | 12.0 |
|  | D-Mannose | 1.5 | 18.0 |
|  | 1-Tetradecanol | 0.5 | 6.0 |
|  | *myo*-Inositol | 1.5 | 18.0 |
| Total | — | 10.0 | 120.0 |
| Formulation 2 | *n*-Butylamine | 3.0 | 36.0 |
|  | Succinic acid | 3.0 | 36.0 |
|  | L-Pyroglutamic acid | 1.5 | 18.0 |
|  | L-Leucine | 1.5 | 18.0 |
|  | Syringic acid | 1.0 | 12.0 |
| Total | — | 10.0 | 120.0 |

**Table S2** Individual indicator scoring scale for sensory quality characteristics of tobacco leaves

| Indicator | Score range | | | | |
| --- | --- | --- | --- | --- | --- |
|  | <5.5 | [5.5-6.0） | [6.0-6.3） | [6.3-6.5） | ≥6.5 |
| Aroma quality | Poor | Slightly poor | Fair | Good | Excellent |
| Amount of aroma | Thin | Slight | Fairly sufficient | Quite sufficient | Rich |
| Concentration | Heavy | Slightly heavy | Present | Slight | Very slight |
| Irritancy | Strong | Slightly strong | Present | Slight | Very slight |
| Offensive odor | Uncomfortable | Slightly uncomfortable | Fairly clean & comfortable | Quite clean & comfortable | Clean & comfortable |
| Physiological strength | Poor | Slightly poor | Fair | Good | Excellent |
| Aftertaste | Small | Slightly small | Large | Slightly large | Moderate |
| Softness and fineness | Poor | Slightly poor | Fair | Good | Excellent |
| Sweetness | Poor | Slightly poor | Fair | Good | Excellent |

Note: The indicators refer to the sensory quality evaluation method of Hebei China Tobacco and the “2025 Guizhou Tobacco Leaf Quality Fixed-point Monitoring Report”.

**Table S3** Mean sensory scores and standard deviations of cured middle tobacco leaves under different fertilization treatments.

| Indicator | Treatment | | | | | |
| --- | --- | --- | --- | --- | --- | --- |
|  | CK1 | CK2 | A1 | A2 | A3 | A4 |
| Aroma quality | 6.0 ± 0.10 | 6.1 ± 0.10 | 6.2 ± 0.07 | 6.0 ± 0.10 | 6.4 ± 0.07 | 6.2 ± 0.10 |
| Amount of aroma | 6.0 ± 0.10 | 6.0 ± 0.07 | 6.1 ± 0.10 | 6.0 ± 0.07 | 6.2 ± 0.10 | 6.2 ± 0.07 |
| Concentration | 5.8 ± 0.07 | 6.0 ± 0.10 | 6.1 ± 0.07 | 5.8 ± 0.10 | 6.2 ± 0.07 | 6.2 ± 0.10 |
| Irritancy | 6.1 ± 0.10 | 6.1 ± 0.07 | 6.2 ± 0.10 | 6.1 ± 0.07 | 6.4 ± 0.10 | 6.2 ± 0.10 |
| Offensive odor | 6.0 ± 0.07 | 6.1 ± 0.10 | 6.2 ± 0.07 | 6.0 ± 0.10 | 6.3 ± 0.10 | 6.2 ± 0.10 |
| Physiological strength | 5.8 ± 0.10 | 6.0 ± 0.07 | 6.0 ± 0.10 | 5.8 ± 0.10 | 6.0 ± 0.10 | 6.0 ± 0.10 |
| Aftertaste | 6.0 ± 0.07 | 6.0 ± 0.10 | 6.2 ± 0.10 | 6.0 ± 0.10 | 6.3 ± 0.10 | 6.2 ± 0.07 |
| Softness and fineness | 6.1 ± 0.10 | 6.1 ± 0.10 | 6.2 ± 0.10 | 6.1 ± 0.10 | 6.3 ± 0.07 | 6.2 ± 0.10 |
| Sweetness | 6.0 ± 0.10 | 6.0 ± 0.10 | 6.1 ± 0.10 | 6.0 ± 0.07 | 6.2 ± 0.10 | 6.1 ± 0.07 |

**Table S4** Primers used for qRT-PCR validation

| Gene ID | Forward primer (5′–3′) | Reverse primer (5′–3′) |
| --- | --- | --- |
| LOC107776311 | TTCCGGTGTTGTCACCCTAAA | GGCGAATAAATAATCTCTTGTTGGG |
| LOC107777754 | TTGGGAAGAGTACTGCAATCAG | TCCCTTTGTTCCGTTCCTCA |
| LOC107804321 | ATGAGAGCTGTTGGCAGAGC | GTGTTTCGGGGTCTAACCCTT |
| LOC107812742 | CTGCATGGTAGCATCCTTTGC | TCCATCAAGTTTGTGCATCACC |
| LOC107789474 | GGCTTAGCATGCCTGTTTGG | GCGCTTAAAACCACGTCACA |
| LOC107824990 | AATCGGGAGCTTTTTGTGCC | ACACATGAAGCGAAGGTGGA |
| LOC107814442 | TCCCTCTCTCTTCCGTCTACTG | GCAGCTAATTCCCTCCCGAA |
| LOC107796072 | CGAGGACTTTTGCCCCAAGA | TTTTTCAGTCAGCTTGCCGC |
| Actin | ACCTCTATGGCAACATTGTGCTCAG | CTGGGAGCCAAAGCGGTGATT |

**Fig. S1** Numbers of differentially accumulated metabolites in different fertilization treatment comparisons

**Fig. S2** Venn diagram showing shared and unique differentially accumulated metabolites among different fertilization treatment comparisons

**Fig. S3** Venn diagram showing shared and unique differentially expressed genes among different fertilization treatment comparisons

**Fig. S4** Selection of the soft-thresholding power for WGCNA network construction

**Fig. S5** Heatmap of the gene co-expression network based on the topological overlap matrix

**Fig. S6** Pearson correlation heatmap of phenylpropanoid–flavonoid pathway-related genes and metabolites in fresh middle tobacco leaves under different fertilization treatments.

**Fig. S7** qRT-PCR validation of selected candidate hub genes

(a–e) Expression comparisons of eight candidate hub genes among CK2 vs CK1, A1 vs CK1, A2 vs CK1, A3 vs CK2 and A4 vs CK2, showing generally consistent trends between RNA-seq and qRT-PCR.
